# Joyful and serious intentions in the work of hospital clowns: A meta-analysis based on a 7-year research project conducted in three parts

**DOI:** 10.3402/qhw.v8i0.18907

**Published:** 2013-01-04

**Authors:** Lotta Linge

**Affiliations:** School of Social and Health Sciences, Halmstad University, Halmstad, Sweden

**Keywords:** Meta-analysis, hospital clowns, child perspective, quality of medical care, humor attunement, magical attachment, joy without demands, transitional area, vicarious therapeutic clown figure

## Abstract

The present meta-analysis focuses on a 7-year research project entitled “Hospital clowns—in encounters with ailing children” and funded by the Swedish Childhood Cancer Foundation. The aim of the meta-analysis, which is based on the project's three studies, was to attempt to achieve a deeper psychological and more nuanced understanding of the unique encounters taking place between the hospital clowns and ailing children in the study. The methodological procedures were qualitative and included 51 interviews with four informant groups: the clowns, staff, children, and their parents. The meta-analysis revealed the unique aspects of hospital clowns’ work with respect to: a) a quality of care that transcends boundaries, that is, a magical safe area where demands and adjustment were temporarily set aside and where the lighter side of life took precedence; b) a non-demanding quality of care, where joy could be experienced without requiring something in return, where the child's terms mattered and where the child perspective was clearly in focus; and c) a defusing quality of care, which is expressed as a positive counterweight that was otherwise lacking in medical care, where the hospital clowns used different solutions that bypassed regular hospital routines by temporarily distracting and making things easier for the children, parents, and staff in various care situations. Finally, the aim of the theoretical framework, in its synthesizing form, was to promote further psychological understanding of the area of humor that exists between fantasy and reality—an intermediate or transitional area that the hospital clowns created together with the children. In this transitional area, the hospital clowns’ unique contribution can be interpreted, in psychological terms, as being available as *a* vicarious therapeutic clown figure in a magical world that parallels reality.

The clown figure, with its humorous profile and colorful clothing, elicits many different reactions among children and adults. Carp (1998) considered various clown figures and pointed out “the trickster, the fool and the clown” as archetypical images. The trickster is often presented as a symbolic representative of an animal, the fool as a marginal figure in society, one without social norms, and the clown as a circus clown or performer. McGhee and Goldstein (1983) wrote: “Whether one sees the circus clown as mediating between cultural order and natural disorder, between the adult and the child, or between joy and sadness, there is something about the clown's behavior that is both realistic and absurd” (p. 162).

The clown role can serve several purposes. The hospital clown works at a hospital and wishes to give ailing children both experiences of joy and opportunities to be creative in the context of play. His/her intention is to stimulate the healthy part of the child's being and to mitigate the effects of a hospital stay.

In the international research conducted on hospital clowns in the care of ailing children, studies have shown how clown encounters can have positive effects on children, parents, and hospital staff. For instance, Battrick, Glasper, Prudhoe, and Weaver (2007) showed that the majority of the children in their study experienced joy in the company of hospital clowns. Even the children's parents stressed the importance of the presence of clowns at the hospitals. Weaver, Prudhoe, Battrick, and Glasper (2007) showed how clown humor can mitigate the negative effects of hospital visits for children between 4 and 11 years of age. Her final report from The Clown Doctor Project, Marcon (2005) revealed how well-being can increase among children, parents, and staff. Vagnoli and co-workers (2005, 2010), Golan, Tighe, Dobija, Perel, and Keidan (2009) as well as Fernandes and Arriaga (2010) have illustrated the value of hospital clown encounters and how the clowns can help to relieve not only young children's but also their parents’ anxiety prior to surgery. Hansen, Kibaek, Martinussen, Kragh, and Hejl (2011) emphasized the fact that repeated hospital clown encounters are of more significance than encounters that occur less frequently. They also took a gender perspective by considering whether the clown is male or female and whether and how gender impacts the relationship with the children.

Although the research primarily presents the value of hospital clowns’ work in care and the positive power of humor, there is also research showing the more complex aspects of humor. Ruch (2012) suggested that “humor” is a neutral and catchall concept that can also have negative undertones. Platt and Ruch (2009) focused on the phenomenon of gelotophobia, which refers to the fear of being laughed at. They discussed various degrees of gelotophobia, from milder to more severe forms. It was Titze (2009) who coined the term gelotophobia, calling it a variant of anxiety that is tied to shame. Ruch (2009) discussed the causes and consequences of this type of anxiety and referred to a model presented by Titze (2009), who suggested that behind such anxiety may lie traumatic experiences of having been laughed at as a child or adult, or of not having been treated in a serious and respectful manner. This kind of profound fear of not being taken seriously may ultimately lead to a distrust of expressions of joy and spontaneity in various social contexts.

At present, there are hospital clowns established at most university hospitals in Sweden. Hospital clown activities began in the mid-1990s in Gothenburg with only a few clowns, and this increased in the early 2000s to the present 60 or so clowns working at different hospitals throughout the country. Most hospital clowns work part-time and are not employed by the medical care system. Their working methods vary across the country, as do their ties to the medical care system. In 2006, the Swedish Childhood Cancer Foundation agreed to fund a 7-year research project “Hospital clowns—in encounters with ailing children”, which has been conducted in southern and central Sweden. The focus has been on four informant groups: hospital clowns (Study 1: 2006–07); care-givers (Study 2: 2008–09); children (Study 3a: 2009–12), and parents (Study 3b: 2009–12). These studies have been both empirical and theoretical, and have resulted in three articles published in international journals (Linge, 2008, 2011, 2012a), and three articles published in Swedish journals (Linge, 2007, 2010, 2012b).

The aim of the meta-analysis, which is based on the project's three studies, was to attempt to achieve a deeper psychological and more nuanced understanding of the unique encounters taking place between the hospital clowns and ailing children under the study. The specific questions that comprise the starting point of the present meta-analysis are: What psychological features are observed that can reveal the unique qualities of care found between the hospital clowns and the children? In what way can an overall theoretical framework promote a deepened psychological understanding of the humorous process taking place between the hospital clowns and the children?

## Method

In order to achieve a deeper and more nuanced understanding of the hospital clowns’ encounters with ailing children, a meta-analysis has been conducted on the three studies included in the 7-year research project. The concept meta-analysis, described in the book *Meta-study of Qualitative Health Research* (Paterson, Thorne, Canam, and Jillings 2001), refers to research involving a comprehensive analysis of a number of studies dealing with a specific phenomenon. Methods, theories, and results are presented with a view to creating new insights and new ways of thinking about the phenomenon through a process of synthesis. Paterson et al. (2001) wrote:

Meta-study is composed of four distinct components: the analytic components of meta-data-analysis, meta-method, and meta-theory, and the synthetic component of meta-synthesis. The additional research processes within which these components are contextualized are formulation of the research questions(s); selection and appraisal of data from primary research; and dissemination of the findings of meta-study. Taken together, these analytic processes are part of a comprehensive research approach to provide breadth and depth to the examination and understanding of the phenomenon of concern. (pp. 13–14)

### Sample

The project has employed a qualitative design using interviews (1–1.5 h each) as its methodological approach. Interview data have been gathered from a total of 51 individuals in southern and central Sweden, allowing a detailed analysis of the hospital clowns’ activities.

#### Study 1 (2006–2007): n=13 hospital clowns

In 2006, the hospital clowns worked on a number of wards (oncology, orthopedics, and general medicine). The clowns often worked in different pair constellations, with the exception of one clown who worked as a silent hospital clown with a play therapist.

#### Study 2 (2008–2009): n=20 hospital staff

The staff had all met the hospital clowns from Study 1, which was a criterion for participation. The staff group included representatives at the management level (senior physician) as well as ward level (registered nurses and other nursing staff). The wards (oncology, orthopedics, and general medicine) were the same as those included in Study 1.

#### Study 3a (2010–2012): n=9 children (3–18 years)

The participating children had all been admitted to the hospital for various lengths of time on the three wards (oncology, orthopedics, and general medicine). All had met the hospital clowns from Study 1, which was a criterion for participation. Two of the children were siblings; both had met the clowns previously.

#### Study 3b (2010–2012): n=9 parents

The participating parents had all been with their child on the wards. All had met the hospital clowns from Study 1, which was a criterion for participation.

#### Follow-up study (2012): n=1 hospital clown

To further our understanding of the work of hospital clowns, and considering the future of their efforts, a follow-up interview (1.5 h) was conducted with one of the clowns from Study 1. This particular hospital clown was involved in starting hospital clown work in Sweden and has considerable experience and knowledge, dating back to the early 1990s. The thoughts that emerged from this interview are presented in the discussion section.

### Material

The following themes in each study were used as a framework for the semi-structured interviews:

#### Study 1/hospital clowns

Working methods, personal driving forces, relations to the children, important events, obstacles and possibilities, position in the organization and the value of one's own work.

#### Study 2/staff

Working methods, staff members’ work roles, cooperation with hospital clowns, the children's well-being, obstacles and possibilities, and the value of hospital clowns’ activities.

#### Study 3a/children

Encounters with the hospital clowns in the context of care, working methods, significance, obstacles and possibilities, the value of clown encounters, and desires concerning future hospital clown activities. In meetings with the youngest children, open interviews were used, allowing the children to tell about their encounters with hospital clowns entirely on their own terms.

#### Study 3b/parents

Encounters with hospital clowns in the context of care, working methods, importance for the child and the parents, limitations and possibilities, the value of clown encounters, as well as desires concerning future hospital clown activities.

#### Follow-up interview/hospital clown

Future development of hospital clown work.

### Procedures

All data from Study 1–3 (2006–2012), the follow-up interview (2012), as well as the six articles published in Sweden and in international journals have been included in the present meta-analysis.

### Qualitative analysis

Paterson et al. (2001) wrote: “Meta-study is composed of four distinct components: the analytic components of meta-data-analysis, meta-method, and meta-theory, and the synthetic component of meta-synthesis” (pp. 13–14).

These four components referred to by Paterson et al. can be explained as follows: 1) In the present meta-analysis, meta-data-analysis refers to a rereading of the entire corpus of data in order to achieve an overall picture and a deeper understanding of the data, focusing on what is unique in terms of the qualities of care; 2) Meta-method-analysis refers to an analytical procedure in line with Interpretative Phenomenological Analysis, IPA (Eatough & Smith, 2008; Shinebourne, 2011). The work involved in the meta-method-analysis was to identify possible main themes and associated sub-themes in the three project studies. Using this approach, three main themes emerged that reflected the content of the three project studies, namely a quality of care that transcends boundaries, a non-demanding quality of care and a defusing quality of care; 3) Meta-theory-analysis concerns a critical examination of the theoretical interpretations made in the three studies, in line with Tomkins’ (1962, 1963, 1991) affect theory as well as with Bowlby's (1988) and Ainsworth's (1978) attachment theories, the aim being to arrive at an overall theoretical approach. McKenna (1997) mentioned “theory framed research”, where additional theory construction is used as a theoretical framework to achieve a comprehensive deepening of the whole in the data; and 4) Meta-synthesis refers to the development of the following theoretical concepts “transitional area” and “transitional relatedness” (in line with Winnicott, 1951, 1971; object relations theory) and the new psychological concept “vicarious therapeutic clown figure”. Thus, the present purpose to offer a deeper psychological understanding of a magical intermediate or transitional area, where the hospital clown, acting as a vicarious therapeutic clown figure, relate to the children and have as his/her mission to bind the inner mental world with the various demands of the external reality. Paterson et al. (2001) wrote: “… meta-synthesis takes the insights developed to an new level of awareness and creates the possibility of stronger more complete, or more theoretically responsible ways of understanding or interpretation” (p. 122).

### Ethical considerations

The local ethics committee of Halmstad University, Sweden, approved the study design (reg. no.: 90-2008-486), as did the regional ethics board at Lund University, Sweden (reg. no.: 2009/357).

## Results

### Qualities of care

The present meta-analysis want to present three qualities of care, that reflect what is unique psychological values, about the work of the hospital clowns: a quality of care that transcends boundaries, a non-demanding quality of care, and a defusing quality of care.

#### A quality of care that transcends boundaries

The expression “a magical safe area” can be used to represent a psychological quality of care and as a metaphor for the phenomenon of clown encounters in the care of ailing children. A magical safe area represents a quality of boundary transcending creative possibilities, where demands and adjustment are temporarily set aside to allow a different magical reality to emerge. The children in particular talked about this safe area, this place between fantasy and reality, as a magical room—a way station of creative possibilities where inner needs and desires could be made visible and where the focus was on the healthy rather than ill sides of the child. The hospital clowns stressed “the lighter side of life,” which runs parallel to the darker side. This safe area can be seen as a resting place in the present moment, where one can temporarily forget one's difficulties in a magical relation with the hospital clowns, and where one can test one's own pleasurable fancies. Here, the *child perspective* is particularly clear, in that the hospital clowns’ intention is to encourage the child to express his/her own wishes. This is in great contrast to the regular situation in medical care and treatment, where in most cases the desires of the adult world must take precedence.

Linge (2012a) called this magical relation “magical attachment” (with reference to the theories of Bowlby, 1988, and Ainsworth, 1978), which is achieved through the hospital clowns’ different approaches to relating to and working with children. Most clowns worked in pair constellations, first interacting with each other to help the child get used to the unusual encounter and to allow the child to interact at his/her own pace and on his/her own terms. The hospital clown who worked as a silent clown followed all of the child's bodily intentions in a synchronized “dance of reciprocity”. Such an approach allows, e.g., immigrant children or children with communication difficulties to actively control the situation and to creatively identify a specific body language that enables them to communicate and fulfill their wishes. The establishment of *reversed roles*, in which the children are strong and brave and the clowns clumsy and weak, also gives children an advantage and helps increase their confidence in their own power of initiative.

#### A non-demanding quality of care

The possibilities of joy can be highlighted as a psychological quality of care, here in terms of “joy without demands”, where laughter is in most cases a clear marker. This kind of joy is free from obligations, requires nothing in return and does not demand adjustment. To acquire a deeper understanding of the possibilities and complexity of joy (Linge, 2011), a theoretical interpretation based on Tomkins’ (1962, 1963, 1991) affect theory is useful. Tomkins (1962) discussed the affect surprise, the task of which is to first reset the nervous system, in order to make way for a new affect, for instance interest, in the hope that the affect joy will finally result in the lingering affect contentment. Part of the hospital clowns’ well-considered working method is the strategy of capturing the children in a state of surprise over the new situation, in order to then create an interest in the present, where the children's power of initiative is in focus, only to finally pave the way for joy in a pleasure-filled fellowship—joy without demands, obligations or the need for adjustment. This positive affect sequence, a kind of affect-regulation through humor (in line with Nelson, 2012) makes room for the healthy and powerful parts of the child's being, including the sibling's being. Nelson (2012) wrote: “Sensitive adults, siblings and playmates are often successfully able to use laughter, humor, or comedy to soothe a negatively aroused child, enabling children to learn to use it themselves or in relationships with other negatively aroused adults or children” (p. 104). This positive affect sequence can also remain as a specific happy memory immediately after the encounter or as a positive memory that can sometimes last for years and be rendered anew at follow-up visits.

#### A defusing quality of care

The expression “a positive counterweight” can be used to represent an additional quality of care. The staff talked about the clowns’ work as providing a positive distraction that play down medical care and that offers different solutions in hospital situations that are difficult for the child. A main thread in the interview data is how important it is that joy can be the child's focus for a while. Here, the hospital clowns offer the lighter side of life, free from demands and obligations, while the staff stand for routines and treatments that require adjustment on the part of the child. In most cases, children's hospital experiences entail a feeling of not having control and sometimes of powerlessness; it is the adults who make decisions about treatment and care. Paradoxically enough, it is this alternation between two extremes (routines/external control–clown encounters/internal control) that can serve to defuse the situation for the children. This probably leads to a toning down of anxiety prior to treatment, where the children feel they are affirmed and seen and thereby have access to internal control when expressing their own will, without the risk of coming out the loser, in a relational sense. Staff described the hospital clowns’ working methods as “a dimension that is lacking in medical care,” “a dimension that increases the quality of the workplace”, and they felt that “the clowns were definitely a departure from our regular routines”. This could conceivably lead to time saving, given that the clowns help the staff by providing a distraction and making preparation for treatment and surgery easier to deal with.

The intention of the present meta-analysis has been to promote a deepened understanding of what takes place during clown encounters and to point out what is unique about them with regard to psychological qualities of care. Finally, the intention of the present theoretical interpretation, in its synthesizing form, has been to promote further psychological understanding of the area of humor that exists between fantasy and reality—an area that the hospital clowns create together with the children. Using the concepts “intermediate/transitional area” and “transitional relatedness” (Winnicott, 1951, 1971) and the new concept “vicarious therapeutic clown figure”, the clinical term quality of care is placed within an overall theoretical framework, in line with “theory framed research” (McKenna, 1997).

## Discussion

### Between fantasy and reality

Winnicott (1951, 1971) talked about an *intermediate* or *transitional area*—an area between fantasy and reality—where an illusion can be maintained during play and where reality can be created and later recreated to suit the child's own wishes and needs. According to Linge (1993), the possibilities of illusion include humor as a tool for creating the unexpected, the absurd, the exaggerated and the paradoxical. Humor can be seen as a synthesizing function, whereby several apparently conflicting forces found in our inner mental world can be brought into harmony with the various demands of our external reality. With the help of hospital clowns, the sly and humorous can take precedence, health problems can be put aside and the child can have a moment of rest and recovery in the present situation.

In this intermediate area, the hospital clowns’ unique contribution can be interpreted, in psychological terms, as being available as *a vicarious therapeutic clown figure* in a magical world that parallels reality. A vicarious therapeutic clown figure, in the form of a hospital clown, can be considered to have a therapeutic role in his/her work with ailing children, but cannot be seen as a therapist in the traditional sense of providing therapy, which is marked by clear time frames and established work contracts. Carp (1998) discussed the therapeutic clown in the context of adult therapy, in which the therapist and the client together test the clown's various role patterns, the goal being to elicit spontaneity and playfulness, use the body as a means of expression, increase the client's tolerance for paradoxes, gain access to unconscious content and, thereby, increase the client's hope for recovery. The present study, focused on ailing children, has shown how a vicarious therapeutic clown figure, in the form of a hospital clown, can change positions (from that of adult to child) and in this way mirror the child's various emotions through *transitional relatedness*. The child is allowed to “bide his/her time in a waiting position” and see a course of events in which two hospital clowns, working in tandem, perform a drama, which actually reflects the child's own problems, before the child himself/herself is able to confront his/her feelings and deal with the different demands of reality. The child can also test the position of being strong and brave, which entails being able to help the weak and awkward clown. The child is thus strengthened in his/her feeling of being active and strong, instead of being the passive and weak party, which should have effects that transfer to the child's ability to deal with his/her own internal problems.

Vicarious therapeutic clown figures express their empathy and show through their attitudes and behavior that the child as well as all his/her affects in this exposed illness situation are understood and respected. *Humor attunement* (Linge, 2008) is the concept that mirrors this relational process, where the hospital clowns empathically tune in to an ongoing affect, shelter it and finally return it to the child in a manageable and humorous form. The goal of a clown encounter is not always to have fun, but for the child and his/her entire emotional register to be seen and acknowledged, with all the affects. What is unique in this context is that the clown encounter is entirely on the child's terms and helps him/her see new creative possibilities, in line with Nelson (2012) and the phenomenon of *affect-regulation*. This, in turn, reinforces the child's self-confidence and belief that he/she can influence the inner affects and can control the course of events in a more humorous way.

One unexpected finding from one of the studies should also be mentioned, namely the often anonymous and invisible position of *siblings*, who find themselves at the periphery of a difficult family situation. Because all the attention is directed at the ailing child, this group of children—the siblings—is most often neither seen nor heard. With the help of hospital clowns, a sibling can also participate and be acknowledged on the same terms as all of the children involved in the joyful play. Such a play situation, free from demands, constitutes a “place to rest in the joy” for a sibling as well—a joy without obligations and demands for adjustment requiring the sibling to always be healthy and well behaved.

### Values and limitations

The present meta-analysis provides strong support for the value of hospital clowns’ work in the context of care. Laughter seems to serve as an indicator that there is a visible healthy side of everyone involved and that joy in the company of the hospital clowns promotes a feeling of well-being. Both the staff and parents expressed their appreciation that they, too, were seen and acknowledged in the clown encounters, and reported that this gave them a moment of rest and recovery from all the difficulties of the entire hospital situation. All of the informant groups stressed the value of the various clown encounters and pointed out their importance, primarily for the children, but also for parents, siblings and staff. The findings of the present studies support results from other research by Battrick et al. (2007), Weaver et al. (2007) as well as from The Clown Doctor Project, where Marcon (2005) revealed in the final project report how the well-being of children, parents and staff can increase following clown sessions.

Questions do arise from the findings, such as how should we view the positive results found in the meta-analysis? Do they apply universally, or are there also certain limitations in both the relational encounter and in various organizational contexts?

Another question that needs to be asked is whether a humorous atmosphere always occurs. According to Ruch (2012), if we view “humor” as a neutral and catchall concept, then it must involve neutral, positive and negative elements. The humorous message may be wrong for the child, the humorous atmosphere may be frightening, the sender and receiver may be out of phase with each other, and developmental stages and personality may determine whether or not the clown encounter feels relevant. The meta-analysis does provide a few examples where parents see a young child's fear of the clowns, where the child avoids the clowns after a difficult treatment and where young teenagers are angry and disappointed when they feel the clowns have treated them in a childish manner. Even though the meta-analysis only reveals a very few examples of inappropriate or lacking sensitivity on the part of the hospital clowns, these examples show the need for an ongoing discussion, throughout the hospital clown enterprise, focused on identifying, early on, any uncertainties that may occur during the clown sessions, the aim being to safeguard the integrity of both the children and their parents.

Another question that needs to be asked in this context is whether the positive affect sequence surprise-interest-joy (Tomkins, 1962, 1963, 1991) always occurs with humorous overtones in the magical encounter between children and hospital clowns. Is it possible that fear and anxiety, too, are sometimes experienced by children and/or parents during clown sessions? Weaver et al. (2007), Vagnoli et al. (2005, 2010), Golan et al. (2009) as well as Fernandes and Arriaga (2010) have shown how hospital clowns, during clown encounters, are able to soothe young children's and parents’ anxiety prior to an upcoming operation, and this finding is supported in the present studies. It should be mentioned, however, that a single child or parent may, on occasion, display a fear of being “laughed at” during a clown encounter. Platt and Ruch (2009) called this phenomenon, i.e., the fear of being laughed at, gelotophobia and discussed the various degrees of gelotophobia. Titze (2009) saw it as a variant of anxiety that is tied to shame. Underlying the condition may be traumatic experiences of having been laughed at or not treated seriously, which in turn may lead to a distrust of expressions of joy and spontaneity in various social contexts. Thus, it is of utmost importance that hospital clowns be sensitive to children's and parents’ personalities and possible social reserve.

An additional question is whether “the positive counterweight” that hospital clowns are supposed to provide in the care situation is always realized. It is likely that working methods, times and conditions as well as employment circumstances vary across cities and hospitals in Sweden. For instance, Hansen et al. (2011) revealed the complexity of the process, indicating that temporal aspects of clown encounters (frequency and duration) may affect how children experience the clowns. Gender too may play a role, in terms of the clowns being male or female, particularly in encounters with teenagers. The present results show, however, that in places where a clown enterprise has been underway continuously for a longer period, those involved seem to have found appropriate methods and times for various clown efforts on the wards. This also applies to places where staff have been open about sharing information on the children's health status as well as information on individual children and parents who sometimes do not wish to have contact with the clowns. Thus, what is important here is showing respect for the desires of each family. Certain phases of the child's treatment may be particularly stressful, and during such phases clown contact involving great creative intensity may be experienced as inappropriate.

One suggestion for *a future central coordination function* for all hospital clowns in Sweden emerged from the meta-analysis. With such a function, the work of hospital clowns could be developed even further, by providing common guidelines for measures to increase competence in the areas of pedagogy, developmental psychology, drama, improvisation, music, and artistry. Discussions on organizational improvements, e.g., sharing experiences among colleagues, establishing mentors for new clowns and promoting new approaches to collaboration with other staff, could provide the hospital clown enterprise with several new creative possibilities. Moreover, providing information for the public should also be seen as an important aspect in terms of justifying the existence of hospital clowns and illustrating the relevance of their work.

## Conclusions and implications for practice

The unique aspects of hospital clowns’ work with regard to its practical benefits for medical care can be summarized as follows:

- a quality of care that transcends boundaries, a magical safe area, where demands and adjustment are temporarily set aside and where the lighter side of life takes precedence. With reversed roles, where the child has the upper hand, a magical attachment occurs between the child and the hospital clowns and the child's healthy side become visible. One effect of this creative possibility may be increased self-confidence on the part of the child, which may help him/her find “short cuts” in life, which in turn may promote his/her recovery, give his/her parents hope, and promote favorable collaboration with staff;
- a non-demanding quality of care, with a perceived feeling of joy without demands for something in return, where the child's terms matter and where a child perspective is in clear focus. The positive affect sequence surprise-interest-joy leads to increased well-being and creates a moment of recreation and relaxation for everyone involved; and
- a defusing quality of care, a positive counterweight, a dimension that is lacking in medical care. The hospital clowns use different solutions that bypass regular hospital routines and that can help children and parents by providing distractions and making things easier, which can in turn lead to time savings in the entire work situation.

## Critical considerations

The present meta-analysis has focused on results from a 7-year research project. All of the data examined come from this project, and a single researcher has been responsible for all of the studies and written all of the articles. Given this, there may be a risk of “blind spots” and of an “overly narrow visual field”. Still, the aim of the meta-analysis has been to illustrate what is unique, in terms of qualities of care, about the hospital clowns’ encounters with children at a number of hospitals in southern and central Sweden. The theoretical framework was also intended to provide a deepened psychological understanding of a phenomenon that previous research has not elucidated in the same way. With continued research and perhaps a mapping of Sweden's entire hospital clown profession, focusing on working methods, times and conditions as well as employment circumstances, society could get an overall picture of the role of hospital clowns in the context of care.
